# Ancient settlements as natural heritage sites: the first occurrence dataset on vascular plant species from ancient settlements in the Lower Dnipro region (Ukraine)

**DOI:** 10.3897/BDJ.11.e99041

**Published:** 2023-01-16

**Authors:** Polina Dayneko, Ivan Moysiyenko, Barbara Sudnik-Wójcikowska, Iwona Dembicz, Maria Zachwatowicz, Nadiia Skobel

**Affiliations:** 1 Kherson State University, Kherson, Ukraine Kherson State University Kherson Ukraine; 2 Insitute of Botany of Slovak Academy of Sciences, Bratislava, Slovakia Insitute of Botany of Slovak Academy of Sciences Bratislava Slovakia; 3 University of Warsaw, Warsaw, Poland University of Warsaw Warsaw Poland

**Keywords:** ancient settlements, flora, the Lower Dnipro, nature conservation, grass steppe, vascular plants.

## Abstract

**Background:**

This work is a long-term outcome of an international Ukrainian-Polish teamwork, aiming to assess the role of ancient settlements for steppe conservation and protection. The dataset contains georeferenced occurrences of vascular plant species on 18 ancient settlements (Lower Dnipro, southern Ukraine), collected during the 2015-2020 period. Additionally, to the total species list, the publication presents the taxonomic coverage (according to GBIF Backbone Taxonomy), the frequency classes of occurrences of the total taxa and the floristic differences amongst studied sites. The report also shows the high sozological value of the studied ancient settlements, the high levels of vascular plant species richness and the various means of the plant species protection (according to the Bern Convention, the Red Data Book of Ukraine and regional Red Lists).

**New information:**

This work provides the first occurrence dataset from ancient settlements in Ukraine. The dataset includes 3,210 occurrences of vascular plants recorded during the study period of 2015-2020 conducted in the Lower Dnipro region. As ancient settlements were generally considered as steppe refuges, great attention was paid to the native steppe species, as well as to the rare components of the flora. The dataset includes 1,525 occurrences of steppe species and 87 occurrences of rare species, respectively. The dataset could be useful for further research of ancient settlements` floristic richness, but also analyses and comparison with other objects of cultural origin (e.g. kurgans, hillforts, old cemeteries, forgotten parks, sacred groves etc.).

## Introduction

Ancient settlements of the Lower Dnipro region (also in some archaeological communities called the Minor Scythia region) were once significant man-made settlements with independent trade, handicraft and characteristic defence complex (Hoshkevych 1913, Gavrylyuk and Krapyvyna 2005, Bylkova 2007, Bylkova 2010, Niemtsev 2014, Gavrylyuk and Matera 2016, Biliaieva et al. 2018). The ancient settlements’ history is still unclear, despite the relatively long period since their foundation (3^rd^—2^nd^ century BC). The archaeological material is still not fully analysed and introduced to the scientific community. Moreover, the material is multi-layered—some of the studied ancient settlements were inhabited by different cultures for subsequent centuries (Bylkova 2007). On the way of determining the ethnicity of the population of the Lower Dnipro settlements, two main directions were identified: “late Scythian” with the nomadic Scythian population and “post Scythian”, where the key role in forming settlements belonged to the inhabitants of ancient centres (Gavrylyuk and Matera 2016). Gavrilyuk and Krapyvyna (1999) note that the population of the Dnipro was influenced in both economic and political factors by Olvia`s polis, because, after the Goth invasion (1^st^ century BC), a large part of the population of Olvia was forced to move to the banks of the Lower Dnipro (Gavrilyuk and Krapyvyna 1999). However, today we can rather define the barbaric character of these two cultures separately, than to assume the development of one culture, as professor Bylkova claimed (Bylkova 2007).

Being both archaeological and cultural monuments, ancient settlements are well assured from the government. First of all, the support is expressed in their status of “monuments of national importance”, which was given to all studied sites in the years of 1960-1970. Remarkably, the question of the natural potential of the ancient settlements was not raised in the past or was raised only by botanists (Moysiyenko et al. 2018b). Nevertheless, such unique objects, combining both cultural and natural heritage, need to be widely recognised and should be the subject of further interdisciplinary scientific research.

Such archaeological monuments are surrounded by continuous agricultural landscapes and constitute some of the last steppe enclaves along the Lower Dnipro region. Located on the Dnipro River terrace, usually between two gullies and covered by steppe vegetation, they become the refuges for many steppe species. During our research, we proved the floristic value of 18 ancient settlements of the Lower Dnipro region. We assessed the structure of their flora (Dayneko 2020a, Dayneko 2020b, Moysiyenko et al. 2020), the level of its synanthropisation (Moysiyenko et al. 2020) and the factors influencing the vascular plant species diversity (Dayneko et al. 2020).

Our results confirmed the role of ancient settlements not only as potential refuges of vascular plants, but also for other representatives of biodiversity. For instance, in the Staroshvedsky ancient settlement, the camel spider (*Galeodesaraneoides* Pallas, 1772) was found. The last record of this species from the territory of continental Ukraine was confirmed 100 years ago (Moysiyenko et al. 2018a). Such unique findings emphasise the need for comprehensive analysis and preservation of these archaeological monuments.

## General description

### Purpose

The general floristic value of the ancient settlements of the Lower Dnipro is undisputable (Moysiyenko et al. 2018b, Dayneko 2020a, Dayneko 2020b, Moysiyenko et al. 2020, Moysiyenko and Dayneko 2021). When preparing the occurrence dataset, the species lists were critically reviewed (in turn, some species were merged as a synonym and some were added as a result of the last fieldwork in spring 2020). For example, during the last field research (spring 2020), we noted the presence of a rare species *Jurineacyanoides* (L.) Rchb., listed in the Bern Convention (Council of Europe 1988).

Our main aim is to summarise the occurrence data from ancient settlements (especially the occurrences of steppe and rare components of the flora) and to make them available for the international audience (in order to generate new scientific knowledge and also for further work to assure the better conservation of the natural values of these sites).

## Project description

### Title

Northern Europe 2022

### Personnel

Ivan Moysiyenko, Mats Widgren, Brian Kuns, Sara Cousins, Janken Myrdal, Camilla Eriksson, Olexandr Khodosovtsev, Ihor Pylypenko, Vitaliy Klymenko, Polina Dayneko, Maria Zachwatowicz, Barbara Sudnik-Wójcikowska, Iwona Dembicz.

### Funding

The collecting of floristic data, field investigations and data analysis were supported by the project “How the East was won: Towards an environmental history of the Eurasian steppe № 2012-06112”, Swedish Science Council.

Further additional analysis and data publishing were supported by the project: “Impact of war on cultural heritage sites as refugia of biological diversity D596”.

We are also grateful to the “Finish Biodiversity Information Facility” (FinBIF) for their call for authors in the project “Northern Europe 2022”.

## Sampling methods

### Sampling description

The study of each site (in total 18 ancient settlements) was conducted at least three times during the growing season (in spring, summer and autumn).

The abundance of individual species was assessed using a 3-point scale: 1 – sporadic (single occurrence), 2 – infrequent (several localities), 3 – common (quite prelevant species within the site) (Sudnik-Wójcikowska and Moysiyenko 2012).

In addition to the total species list for each ancient settlement, we also provided the multi-scale “biodiversity plots”, according to the EDGG standardised sampling approach for biodiversity data (Dengler et al. 2016). The nested plot sampling, covered plot sizes of 0.0001, 0.001, 0.01, 0.1, 1, 10 and 100 m² and included information on the cover of plant species (%), aspect, inclination, the cover of litter, dead wood, stones and rocks, as well as gravel and fine soil, the maximum height of vegetation, the microrelief, the type of management etc.

To assess the frequency category of the species in the studied sites, the following scale was used: I – rare (< 17% i.e. 1–3 ancient settlements, II – relatively rare (18–34% i.e. 4–6 ancient settlements), III – not rare (35-50% i.e. 7–9 ancient settlements), IV – relatively frequent (51–67% i.e. 10–12 ancient settlements), V – frequent (68–84% i.e. 13–15 ancient settlements), VІ – common (85–100% i.e. 16–18 ancient settlements).

The total floristic list, as well as additional information about each taxon (functional group, species life form, species lifespan, species status in the historical-geographical classification, the number of old settlements on which the particular species occurs and origin in the case of alien species) were presented in our previous works (Moysiyenko et al. 2018b, Dayneko 2020a, Dayneko 2020b, Moysiyenko et al. 2020).

### Quality control

The collected materials were verified in the Herbarium of the Kherson State University (KHER), documented and deposited in the form of herbarium specimens (more than 200 herbarium sheets). For data cleaning used OpenRefine.

### Step description

The following steps were taken:

1. Laborious and extensive work with archaeological data was taken before the field research. In order to compile a general description of the settlements, we used the archaeological literature sources and our own observations and we consulted with archaeologists and historians (Lopushynskyi A, Nemtsev S and Sikoza D in *colloquio*). The general information about the settlements of the Lower Dnipro (archaeological name, position, geographical coordinates, area, distance to modern settlements) has been presented in our former work (Moysiyenko et al. 2020). All well-preserved ancient settlements of the Lower Dnipro region were included in the study.
2. The fieldwork was carried out during the growing seasons of the 2015-2020 period.
3. For each ancient settlement, we prepared a total species list and additional data (functional and geographical-historical groups, frequency classes, species life form and lifespan etc.). In addition to floristic data, we also collected information about the type of management, environmental features etc.
4. The total list of vascular plant species included 525 species and 3,210 occurrences (Dayneko et al. 2023) compiled in CSV files. Data were post-processed using Darwin Core terms (Wieczorek et al. 2012).

## Geographic coverage

### Description

**Figure 1.**
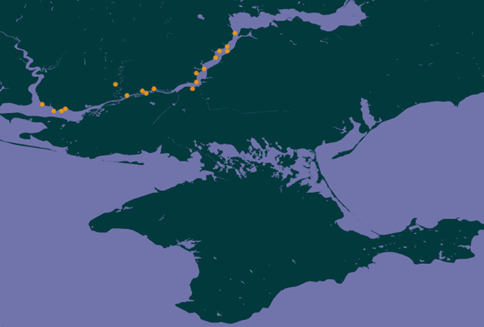
Location map of the studied ancient settlements within the Lower Dnipro region (southern Ukraine).

The research was conducted in the Lower Dnipro region (southern Ukraine), in the Kherson and Mykolaiv Regions (Fig. 1).

The studied area is located within the West Pontic grass steppe zone of the Eastern European Plain (Bohn et al. 2000). The climate is continental with mild winters and long hot summers and low precipitation values (350-420 mm per year). The dominant soils are: low-humus chernozem, dark chestnut, sod and clay sand and meadow-swamp soils (Marynych and Shyshchenko 2005). According to the geobotanical division of the Eurasian Steppe Zone, the Lower Dnipro region is located in the Black Sea and Azov sub-province of the Pontic steppe province (Andrienko et al. 1977).

**Figure 2.**
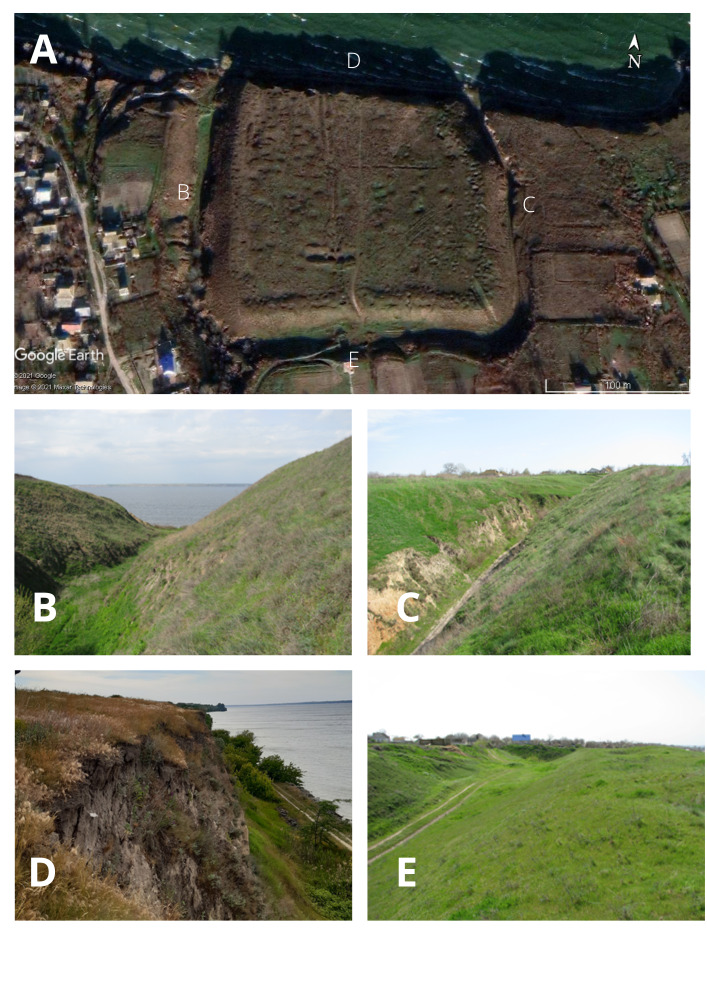
General view of Liubymivske ancient settlement, based on a satellite image from Google Earth Pro (Google Inc 2022) (**A**) and photographs of its boundaries: **B, C** western and eastern (ravines), **E** southern (artificial embankment and a moat), **D** northern (cliff of the Kakhovsky Reservoir) (Photo authors: Moysiyenko I., Dayneko P.).

All 18 studied settlements were located on the steep bank of the Dnipro River on both sides of the river (Kherson and Mykolayiv Regions), usually between two closely-spaced ravines or “balkas” (Fig. 2). An exception was the settlement Oleksandrivka-Roksanovka, which is located on the right tributary of the Dnipro – the Ingulets River. The area of the settlements varies from 1.1 ha (Zolotobalkivske) to 18.7 ha (Velyke Tiagynske).

### Coordinates

47.37 and 46.48 Latitude; 32.00 and 33.97 Longitude.

## Taxonomic coverage

### Description

**Figure 3.**
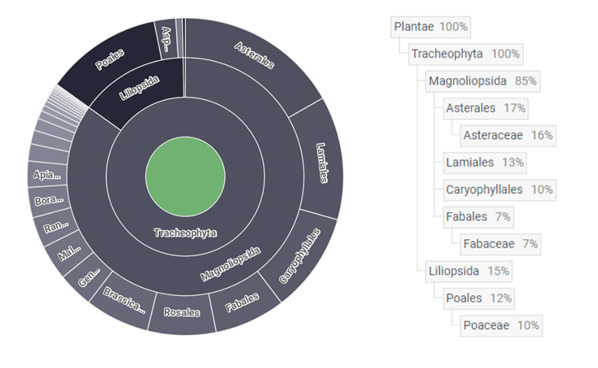
The taxonomic distribution of occurrences within ancient settlements.

According to GBIF Backbone Taxonomy (GBIF Secretariat 2021), our dataset includes 525 taxa of vascular flora, which were identified to species. Dataset includes 31 orders (Apiales, Asparagales, Asterales, Boraginales, Brassicales, Caryophyllales, Celastrales, Cornales, Cucurbitales, Dipsacales, Ephedrales, Ericales, Fabales, Fagales, Gentianales, Geraniales, Lamiales, Liliales, Malpighiales, Malvales, Myrtales, Piperales, Poales, Ranunculales, Rosales, Santalales, Sapindales, Saxifragales, Solanales, Vitales, Zygophyllales) and 69 families (Adoxaceae, Amaranthaceae, Amaryllidaceae, Anacardiaceae, Apiaceae, Apocynaceae, Aristolochiaceae, Asparagaceae, Asteraceae, Berberidaceae, Boraginaceae, Brassicaceae, Campanulaceae, Cannabaceae, Caprifoliaceae, Caryophyllaceae, Celastraceae, Convolvulaceae, Cornaceae, Crassulaceae, Cucurbitaceae, Cyperaceae, Elaeagnaceae, Ephedraceae, Euphorbiaceae, Fabaceae, Fagaceae, Geraniaceae, Grossulariaceae, Heliotropiaceae, Hypericaceae, Iridaceae, Juglandaceae, Juncaceae, Lamiaceae, Liliaceae, Linaceae, Lythraceae, Malvaceae, Moraceae, Oleaceae, Orobanchaceae, Papaveraceae, Plantaginaceae, Plumbaginaceae, Poaceae, Polygonaceae, Portulacaceae, Primulaceae, Ranunculaceae, Resedaceae, Rhamnaceae, Rosaceae, Rubiaceae, Rutaceae, Salicaceae, Sapindaceae, Saxifragaceae, Scrophulariaceae, Simaroubaceae, Solanaceae, Tetradiclidaceae, Thesiaceae, Thymelaeaceae, Ulmaceae, Urticaceae, Violaceae, Vitaceae, Zygophyllaceae) (Fig. 3).

Main changes occurred in orders and families composition of the dataset, while the number of taxa, due to original publication (Moysiyenko et al. 2020), stayed constant. Thus, according to the checklist of vascular plants of Ukraine (Mosyakin and Fedoronchuk 1999), our total number of vascular plant species belong to 281 genera, 74 families, three classes and two divisions (Dayneko 2020b). The dominant division Magnoliophyta is represented by the classes Liliopsida (79 species) and Magnoliopsida (445 species) in the following proportion 1:5.6, respectively. Such a proportion is much more characteristic of the steppe flora (1:4.1-6.2 and more) (Krytskaya 1985, Voronova 2008, Bondarenko 2015) than the proportions of the Ancient Mediterranean flora (1:4-4.5 and more) and the Central European flora (1:2.9-3.6) (Moysiyenko 2011), which corresponds to the position of the ancient settlements of the Lower Dnipro region within the steppe zone.

## Temporal coverage

### Notes

2015-04/2020-09.

## Collection data

### Collection name

Herbarium of the Kherson State University (KHER).

### Specimen preservation method

dried and pressed.

## Usage licence

### Usage licence

Open Data Commons Attribution License

### IP rights notes

This work is licensed under a Creative Commons Attribution (CC-BY) 4.0 Licence.

## Data resources

### Data package title

Flora of the ancient settlements within Lower Dnipro – natural heritage with cultural background

### Resource link

https://doi.org/10.15468/ny3avk

### Alternative identifiers

https://ukraine.ipt.gbif.no/resource?r=ancient_settlements

### Number of data sets

1

### Data set 1.

#### Data set name

Flora of the ancient settlements within Lower Dnipro – natural heritage with cultural background

#### Data format

Darwin Core Archive

#### Download URL

https://www.gbif.org/dataset/89292560-7a4c-4a05-a0b7-7c839f1c252d

#### Description

The dataset includes a table with 32 fields in Darwin Core terms and 3,210 records in it (Dayneko et al. 2023).

**Data set 1.** 

Column label	Column description
occurrenceID	An identifier of a particular occurrence, unique within this dataset. We used the species occurrence numbers according to name of cultural heritage site (Ancient settlements), (Anc.sett.plant.001-Anc.sett.plant.3210).
scientificName	The original names of vascular plants according to Vascular Plants of Ukraine. A nomenclatural checklist (Mosyakin, Fedoronchuk, 1999), but corrected for spelling mistakes using GBIF Species Matching tool (with one exception – see Taxonomic coverage description).
organismQuantity	A number or enumeration value for the quantity of organisms. Estimated according to a 3-point scale: 1 – sporadic, 2 – fairly frequent, 3 – common.
organismQuantityType	The type of quantification system used for the quantity of organisms. We used a 3-point scale.
samplingProtocol	The names of the method used during an Event (Species Shoot Presence).
eventDate	The date-time or interval during which an Event occurred.
basisOfRecord	The method in which data were acquired (MaterialCitation).
geodeticDatum	The geodetic datum upon which the geographic coordinates are given (WGS84).
georeferencedBy	A person who determined the georeference (Dayneko P).
georeferenceProtocol	A description of the method used to determine coordinates (Manual with Google Earth).
recordedBy	A persons who was responsible for recording the original Occurrence (Moysiyenko II, Sudnik-Wójcikowska B, Dembicz I, Zachwatowicz M, Dayneko P).
identifiedBy	A persons who assigned the Taxon to the subject (Moysiyenko II, Sudnik-Wójcikowska B, Dembicz I, Zachwatowicz M, Dayneko P).
coordinateUncertaintyInMetres	The distance (in metres) from the given decimalLatitude and decimalLongitude describing the smallest circle containing the whole of the Location (from 94.9 m to 362.6 m).
geoReferenceRemarks	Notes about the spatial description determination, explaining assumptions made in addition or opposition to the those formalised in the method referred to in georeferenceProtocol (describing the smallest circle containing the whole of the Location (from 94.9 m to 362.6 m).
decimalLatitude	The geographic latitude in decimal degrees.
decimalLongitude	The geographic longitude in decimal degrees.
countryCode	The standard code for the country in which the Location occurs (UA).
country	The name of the country which the Location occurs (Ukraine).
stateProvince	The name of the administrative region of Ukraine in which the Location occurs: Kherson, Mykolaiv.
county	The full, unabbreviated name of the next smaller administrative region than stateProvince (districts).
locality	The specific description of the place. The name of ancient settlement, nearest village.
taxonRank	The taxonomic rank of the most specific name in the scientificName.
kingdom	The full scientific name of the kingdom in which the taxon is classified. In our case, it is always Plantae.
phylum	The full scientific name of the phylum or division in which the taxon is classified. In our case, it is always Tracheophyta.
class	The full scientific name of the class in which the taxon is classified. In our case, it is Magnoliopsida, Liliopsida, Gnetopsida.
order	The full scientific name of the order in which the taxon is classified. (Fig. 3; see also: Taxonomic distribution of occurrences, Dayneko et al. 2022).
family	The full scientific name of the family in which the taxon is classified. (Fig. 3; see also: Taxonomic distribution of occurrences, Dayneko et al. (2022)).
genus	The full scientific name of the genus in which the taxon is classified. (Fig. 3; see also: Taxonomic distribution of occurrences, Dayneko et al. (2022)).
recordedByID	A list (concatenated and separated) of the globally unique identifier for the people responsible for recording the original Occurrence.
identifiedByID	A list (concatenated and separated) of the globally unique identifier for the people responsible for assigning the Taxon to the subject.
associatedReferences	A list of concatenated identifiers publication.
identificationRemarks	Comments about the identification of *Crataegusmonogyna* Jacq. s.l.

## Additional information

### Floristic richness and taxonomic value of ancient settlements within Lower Dnipro region

The dataset includes 525 vascular plant species amongst 18 ancient settlements. As it was mentioned in our previous works (Moysiyenko et al. 2020), the number of species amongst the studied objects ranged significantly from 124 (Gavrylivske) to 290 (Velyke Tiagynske). An average number of occurrences per archeological site is 178 species. The high floristic richness of the studied flora is primarily determined by the spatial location of settlements along the Dnipro River and the ecotopic diversity, in particular, the presence of slopes of various exposures and steepness, rock outcrops, as well as the neighbourhood of settlements with significant steppe areas.

**Figure 4.**
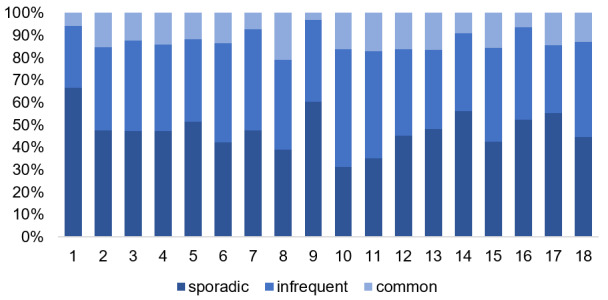
Distribution of species (according to the 3 – point scale of abundance within the studied ancient settlements (in percentages). Explanation: 1 – Chervonomaiatske, 2 – Gavrylivske, 3 – Glyboka Prystan, 4 – Hannivske, 5 – Konsulivske, 6 – Liubymivske, 7 – Lvivske, 8 – Male Tiagynske, 9 – Oleksandrivka-Roksanivka, 10 – Poniativske, 11 – Sablukivske, 12 – Skelka, 13 – Stanislavske, 14 – Staroshvedske, 15 – Velykolepetykhske, 16 – Velyke Tiagynske, 17 – Zolotabalkivske, 18 – Zolotyi Mys.

The distribution of species according to the 3–point scale of abundance differs amongst the studied objects (Fig. 4). Such ancient settlements as Chervonomaiatske, Staroshvedske, Zolotobalkivske, Velyke Tiagynske Konsulivske and Oleksandrivka-Roksanivka are characterised by the dominant role of sporadic occurrences and, at the same time, are the most species rich.

The taxonomic coverage and distribution of dominant families were considered more in detail while the qualitative composition of dominant families, genus etc., as well as the frequency classes of occurrences of total taxa, were missed.

**Figure 5.**
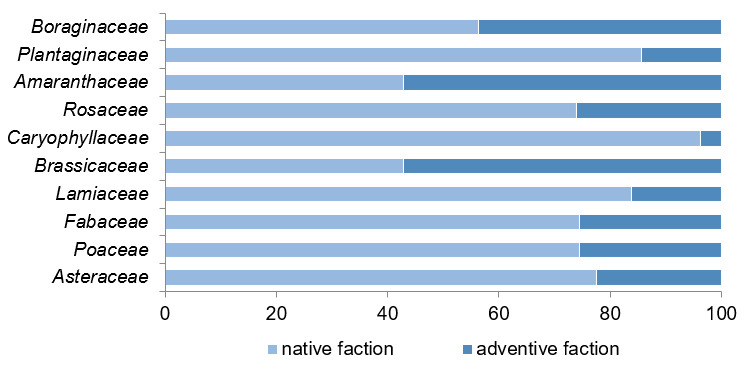
Distribution of occurrences of the dominant families amongst native and adventive fractions of studied flora (%).

Within the total list of studied flora, dominant families have a rather different ratio of native and adventive species in their composition (Fig. 5). The role of local native species in the leading families varies from 42.9% to 96.2%, with an average of 70.9%. The significant differences in the overall range are explained by the shares related to the families Amaranthaceae and Brassicaceae, which both have 42.9% of native species, respectively, while most of the species in their composition are adventive. On the other hand, the family Caryophyllaceae, contains only one adventive species - *Cerastiumtomentosum* L. The species spectrum of this family is represented by the native fraction (96.2%), amongst which, some rare (on the regional level) species occur (*Dianthusandrzejowskianus* (Zapal.) Kulcz. and *Silenesupina* M. Bieb.).

Another indicator of the systematic structure of the flora is the spectrum of its leading genera, which more sensitively reflect the peculiarities of the flora compared to the family spectrum (Novosad 1992). Amongst 281 genera, the most dominant are *Veronica* (13 species), *Astragalus* L. (9 species), *Artemisia* L. (8), *Carex* L. (8), *Euphorbia* L. (8) and *Galium* L. (8).

The complete absence of adventive species from the genera includes *Achillea* L., *Allium* L., *Astragalus* L., *Carex* L., *Dianthus* L., *Euphorbia* L., *Potentilla* L., *Salvia* L. and *Verbascum* L. Such genera as *Allium* L., *Astragalus* L. and *Dianthus* L. are represented entirely by non-synanthropic species. In contrast, the genera *Chenopodium* L. and *Atriplex* L. were represented mostly by adventive species; 57.1% and 66.7% of the adventive fraction, respectively.

Overall, the systematic structure of the total taxa reflects the general zonal characteristic of the Holarctic flora. The most dominant families of Asteraceae, Poaceae and Fabaceae, which make up 30.9% of the spontaneous flora, are characterised by one of the largest shares of the native fraction (74.4-77.6%). The shift in the structure of the flora is largely due to synatropisation processes, in particular, the spread of adventive plants from neighbouring agro-landscapes. This is evidenced by the presence of the Amaranthaceae, Brassicaceae and Boraginaceae families in the family spectrum within the ancient settlement flora. However, the anthropogenic processes are usually limited and extensive in nature, which is confirmed by the dominant role of the native element at all taxonomic levels of the flora.

**Figure 6.**
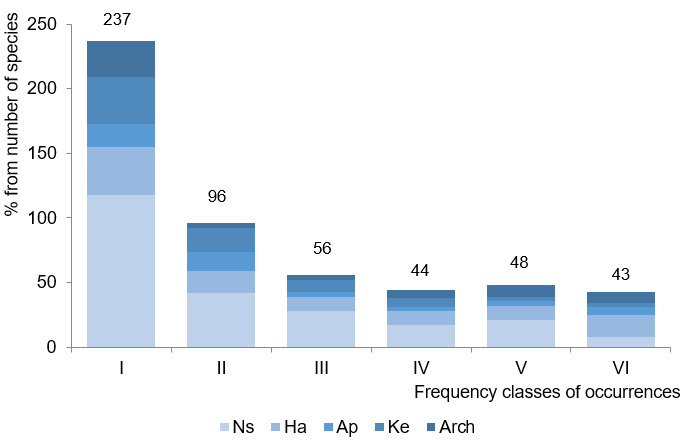
Distribution of geographical and historical groups of the settlements of the Lower Dnipro by frequency classes of occurrences (the absolute number of species in each category is indicated at the top of the bar). Explanation: Ns – non-synanthropic species, Ha – hemiapophytes, Ap – euapophytes, Arch – archaeophytes, Ke –kenophytes.

Regarding the frequency classes of occurrences, 237 species (45.2% of the total taxa) were assigned to the first (I) class of frequency of occurrences and were represented by the species found only on 1-3 ancient settlements (Fig. 6). This is particularly noteworthy as more than 50% of the above-mentioned group consists of indigenophytes, including rare plant species (27 species; or 81.8% of all protected species within ancient settlements). In general, groups of plants of the first and second classes make up 3/4 of the total flora of the settlements of the Lower Dnipro region, which indicates its heterogeneity and uniqueness.

The last (VI) class contains the most widespread species within general taxa, represented by 43 species (8.2%). This group is represented mostly by adventive species, with the exception of *Artemisialercheana* Weber ex Stechm., *Festucavalesiaca* Schleich. ex Gaudin, *Kochiaprostrata* (L.) Schrad., *Koeleriacristata* Kar. & Kir., *Potentillarecta* L., *Teucriumpolium* L., *Thymusdimorphus* Klokov & Des.-Shost. and *Verbascumphoeniceum* L. (Moysiyenko et al. 2020). As a result, the tendency to a decrease in the share of adventitious species to decrease in the class of frequency of occurrence can be observed. This allows us to confirm their limited influence in the flora of these archaeological monuments.

### Sozological value of ancient settlements

The ancient settlements of the Lower Dnipro, along with the nature conservation sites, are characterised by a high representation of steppe and rare Red-listed vascular plants, which emphasises their role as a refuge of dry grasslands (Dayneko and Moysiyenko 2020, Dayneko et al. 2020, Moysiyenko and Dayneko 2021).

Overall, we identified 33 species in the Red List (6.3% of the total number of species), of which: one species is included in the Bern Convention (Council of Europe 1988), 10 species —in the Red Book of Ukraine (Didukh 2009), 21 species—in the Red Lists of the Kherson Region (Kherson Regional Council 2013) and five species—of the Mykolaiv Region (Mykolaiv Regional Council 2021). The total dataset includes 1,525 occurrences of steppe species and 87 occurrences of protected species, respectively.

The sozophytes of the settlements represented three classes, 18 families and 25 genera. Amongst the families, the most numerous is *Poacea* Barnhart (six species), characteristic for the most affected virgin landscapes of the steppe. Other families are represented by only 1-2 species. The leading genera regarding the sozophyte flora are *Stipa* L. (four species), *Astragalus* L. (2), *Jurinea* Cass. (2) and *Tulipa* L. (2).

We showed the highest share of sozophytes and steppe species for the following ancient settlements: Velike Tiagynske, Konsulivske, Skelka and Oleksandrivka-Roksanivka. Such ancient settlements as Glyboka Prystan, Hannivske, Sablukivske and Zolotobalkivske, Velikolepetykhske, Gavrylivske and Stanislavske are noted for their low level of value in terms of the number of sozophytes; however, in terms of the number of steppe vascular plants, these settlements are well represented (Table 1).

The lowest numbers of steppe species (no more than 40% of the total number of species) is noted for Velykolepetykhske, Liubymivske and Male Tiagynske ancient settlements (Table 1). These cultural monuments, with the exception of the last one, are the most anthropogenically transformed objects in many aspects. In the case of Male Tiagynske ancient settlement, such proportion of steppe species can be explained by its isolated location on the Tiagynka River, which contributed to the significant representation of the elements characteristic of the intrazonal floodplain ecosystems.

Evidently, mostly all ancient settlements within the Lower Dnipro region show high conservation value, particularly for steppe ecosystems, but this topic requires further discussion.

## Figures and Tables

**Table 1.** Number of species of steppe and rare protected species within the ancient settlements of the Lower Dnipro.

№	**Ancient settlement**	**Number of protected species**	% **from total number of species**	**Number of steppe species**	% **from total number of species**
1.	Chervonomaiatske	4	2.12	84	44.68
2.	Gavrylivske	1	1.25	54	43.20
3.	Glyboka Prystan	5	3.10	80	49.69
4.	Hannivske	2	1.57	63	49.60
5.	Konsulivske	17	7.11	141	58.99
6.	Liubymivske	1	1.45	52	35.86
7.	Lvivske	3	1.45	92	44.40
8.	Male Tiagynske	3	1.49	78	38.80
9.	Oleksandrivka- Roksanivka	8	3.77	111	52.35
10.	Poniativske	3	2.04	70	47.62
11.	Sablukivske	4	2.53	88	55.69
12.	Skelka	7	3.97	95	53.97
13.	Stanislavske	2	1.28	73	46.79
14.	Staroshvedske	3	1.85	73	45.06
15.	Velykolepetykhske	1	0.56	68	37.98
16.	Velyke Tiagynske	15	5.15	142	48.79
17.	Zolotabalkivske	7	3.61	103	53.09
18.	Zolotyi Mys	1	1.32	58	43.93
	**All settlements**	**33**	**6.28**	**240**	**45.7**
